# Paclitaxel combined with capecitabine as first-line chemotherapy for advanced or recurrent gastric cancer

**DOI:** 10.3892/ol.2014.2131

**Published:** 2014-05-09

**Authors:** MEIQIN YUAN, YUNSHAN YANG, WANGXIA LV, ZHENGBO SONG, HAIJUN ZHONG

**Affiliations:** Department of Chemotherapy, Zhejiang Cancer Hospital, Hangzhou, Zhejiang 310022, P.R. China

**Keywords:** advanced gastric cancer, paclitaxel, capecitabine, efficacy

## Abstract

Chemotherapy is of crucial importance in advanced gastric cancer (AGC) patients, in order to obtain palliation of symptoms and improve survival. To date, no standard chemotherapy regimen has been established for AGC. The purpose of the present study was to evaluate the efficacy and toxicity of the combination regimen of paclitaxel and capecitabine (PX) as first-line chemotherapy in patients with advanced or recurrent gastric cancer. Patients with advanced or recurrent gastric cancer who were treated with PX as first-line chemotherapy between January 2001 and December 2012 at the Zhejiang Cancer Hospital (Hangzhou, China) were retrospectively investigated. Survival was evaluated using the Kaplan-Meier method. In total, 36 patients were enrolled, with a median age of 53.5 years and a Karnofsky performance status (KPS) score of ≥80. A median of 4 PX cycles were administered (range, 2–8 cycles). The median progression-free survival time was 3.7 months [95% confidence interval (CI), 2.9–4.5 months) and the median overall survival time was 12.0 months (95% CI, 9.8–14.1 months). From the 36 patients evaluated, one (2.8%) achieved a complete response, seven (19.4%) achieved a partial response, 24 (66.7%) exhibited stable disease and four (11.1%) exhibited progressive disease. The objective response rate was 22.2% (8/36), and the disease control rate was 88.9% (32/36). All 36 patients were assessed for treatment toxicity. Grade 3 or 4 adverse events included neutropenia (2.8% of patients), hand-foot syndrome (2.8%) and vomiting (2.8%). No neutropenic fever or treatment-related mortalities were observed. PX combination chemotherapy may be a valuable first-line therapy for advanced or recurrent gastric cancer.

## Introduction

Recurrent or advanced gastric cancer (AGC) is one of the leading causes of cancer-related mortalities worldwide (1–3), with a high incidence rate in Asia (4). A large number of patients have unresectable, locally advanced or metastatic gastric cancer at the initial diagnosis, indicating a poor outcome (5). AGC patients have a median survival time of between three and five months, and a five-year survival rate of <10%, if left untreated (6–8).

Previous studies have shown that chemotherapy may improve a patient's survival time and quality of life (6–9). Several types of single drug regimens exist that have certain effects on AGC, including 5-fluorouracil (5-FU), mitomycin, cisplatin and etoposide, with effective rates of 10–20%. In recent years, novel chemotherapeutic agents have included the taxanes (docetaxel and paclitaxel) and oral fluoropyrimidines (capecitabine and S-1), as well as oxaliplatin and irinotecan (10–14). Several studies have analyzed the use of taxanes (paclitaxel and docetaxel) in AGC as single agents or in combination (10,12,13). In V325, a large randomized phase III study, the combination of docetaxel, cisplatin and 5-FU (DCF) was shown to significantly improve the time to progression (TTP), the survival time and the response rate (RR) in untreated AGC patients compared with cisplatin and 5-FU (CF). However, DCF treatment resulted in a certain level of increased toxicity (14). Further studies have demonstrated that paclitaxel plus 5-FU (PF) and docetaxel plus 5-FU (DF) appear to have similar efficacy against advanced or recurrent gastric cancer, with different, but acceptable, safety profiles (15,16). Capecitabine (N4-pentoxycarbonyl-50-deoxy-5-fluorocytidine; Xeloda; Roche Holding AG, Basel, Switzerland) is a 5-FU prodrug developed to reduce the toxicity and enhance the intratumoral concentrations of 5-FU. Capecitabine has been used in preclinical xenograft models, and has been shown to be highly active against several types of tumors, including breast, colorectal, gastric and cervical tumors (17,18), and also against 5-FU-sensitive and -resistant tumors (19). Capecitabine has also been shown to be active in previously untreated AGC patients, as a single agent (20) or in combination with other drugs, including cisplatin, oxaliplatin, epirubicin and docetaxel (20–23). The combination of paclitaxel and capecitabine (PX) in AGC, however, has rarely been reported.

The present retrospective study was conducted to investigate the efficacy and tolerability of the combination of PX in patients with AGC as first-line therapy.

## Materials and methods

### Patients

Patients with advanced or recurrent gastric cancer who were treated with PX as first-line chemotherapy between January 2001 and December 2012 at the Zhejiang Cancer Hospital (Hangzhou, China) were retrospectively investigated. Patients eligible for this study had histologically-confirmed advanced or recurrent gastric cancer. Furthermore, the eligibility criteria included at least one measurable lesion of ≥1 cm in the longest diameter or lymphonodus of ≥1.5 cm in the shortest diameter. Patients were treated with PX as first-line therapy. The study was approved by the ethics committee and institutional review board of Zhejiang Cancer Hospital and conducted in compliance with the Helsinki Declaration.

### Chemotherapy

Paclitaxel (75 mg/m^2^) was administered intravenously for 3 h on days 1 and 8 of the 21-day cycle (or 150 mg/m^2^ on day one of the 21-day cycle), combined with capecitabine (850 mg/m^2^) peroral twice daily on days 1–14. Dose adjustments were made according to the specific situation.

### Adverse effects

Toxicity was measured using the National Cancer Institute-Common Toxicity Criteria version 2.0 (10) toxicity scales. Grade 3 to 4 toxicity was recorded according to the medical records.

### Assessment and statistics

Response was evaluated every two cycles of treatment using the Response Evaluation Criteria in Solid Tumors (24). In cases of partial response (PR) or complete response (CR), a confirmative computed tomography scan was performed four weeks later. CR was defined as the complete disappearance of all evaluable lesions, persisting for four weeks or more. PR was defined as a ≥30% reduction in the sum of the products of the largest perpendicular diameters in all measurable lesions for more than four weeks, without the development of new lesions. Progressive disease (PD) was defined as an increase in a previous lesion by >20%, or the development of any new lesion. Stable disease (SD) was defined as any change in a previous lesion that did not conform with the PR or PD categories. The primary endpoint was progression-free survival (PFS) and the secondary endpoints were overall survival (OS), RR and toxicity. Survival time was analyzed using the Kaplan-Meier software of SPSS version 15.0 (SPSS, Inc., Chicago, IL, USA).

## Results

### Patient characteristics

Due to the exclusion of cases with incomplete data as a result of incomplete medical records and follow-up, 36 patients were investigated between January 2001 and December 2012 at the Zhejiang Cancer Hospital, and their baseline characteristics are shown in Table I. All patients received PX as first-line therapy and among them, 25 were male and 11 were female with a median age of 53.5 years (range, 28–75 years). A median of 4 treatment cycles were administered (range 2–8). In addition, eight patients underwent radical surgery.

### Efficacy

**Figure 1:**
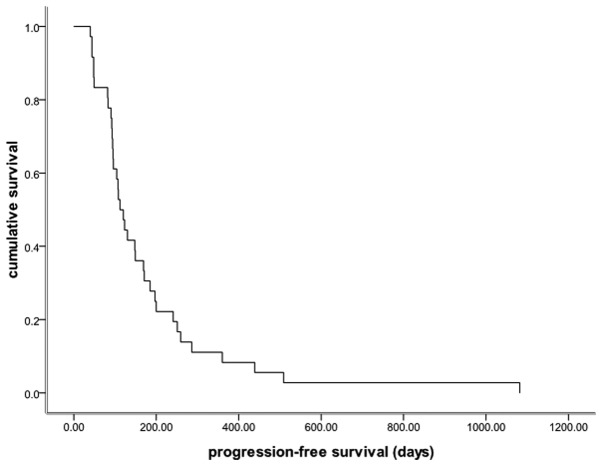
Kaplan-Meier estimates of the PFS time of paclitaxel combined with capecitabine (PX) as first-line chemotherapy for advanced or recurrent gastric cancer. The median PFS time was 3.7 months (95% CI, 2.9–4.5 months). PFS, progression-free survival; CI, confidence interval.

**Figure 2:**
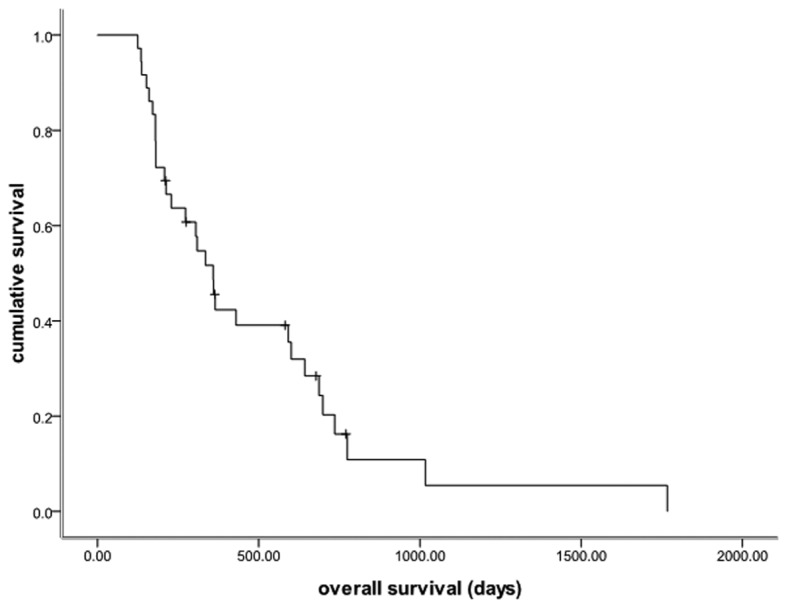
Kaplan-Meier estimates of the OS time of paclitaxel combined with capecitabine (PX) as first-line chemotherapy for advanced or recurrent gastric cancer. The median OS time was 12.0 months (95% CI, 9.8–14.1 months). OS, overall survival; CI, confidence interval.

Out of the 36 patients evaluated, one achieved a CR, seven achieved a PR, 24 exhibited SD and four exhibited PD. The objective RR was 22.2% (8/36) and the disease control rate was 88.9% (32/36). The median PFS time was 3.7 months (95% CI, 2.9–4.5 months; Fig. 1) and the median OS time was 12.0 months (95% CI, 9.8–14.1 months; Fig. 2). The subgroup analysis showed no difference in PFS time, regardless of gender, age, radical surgery and tumor site (Table II).

### Toxicity

All 36 patients were assessed for treatment safety and the main adverse event was found to be hematological toxicity. Grade 3 or 4 adverse events included neutropenia (2.8%), hand-foot syndrome (2.8%) and vomiting (2.8%). No neutropenic fever or treatment-related mortalities were observed. In addition, no other adverse events were recorded in the medical records and no dosage reduction occurred.

## Discussion

The present study retrospectively investigated AGC patients who were treated with PX as first-line chemotherapy between January 2001 and December 2012 at the Zhejiang Cancer Hospital. As few studies have been published concerning PX as first-line chemotherapy in AGC patients, this investigation was significant, however, it was not a prospective study or large-scale randomized trial. In the AGC patients treated with PX as first-line chemotherapy, the objective response and disease control rates were 22.2 and 88.9%, respectively, and the overall median survival time was 12.0 months. These results are comparable to those reported in the study by Kang *et al* (25), with a tumor RR of 48.9%, a median TTP of 5.6 months and a median OS time of 11.3 months. The combination of PX was well tolerated in the present study, with only mild adverse effects [grade 3/4 neutropenia (2.8%), grade 3/4 hand-foot syndrome (2.8%) and grade 3/4 vomiting (2.8%)]. Only one patient was reported with a grade 3 to 4 gastrointestinal reaction, which included nausea and vomiting. Although the gender, age, radical surgery and tumor sites differed between the patients, the subgroup analysis showed no differences between the PFS and OS times.

To the best of our knowledge, AGC patients have a poor prognosis. Thus, treatment for such patients is a critical issue facing medical workers worldwide. Systemic chemotherapy is widely accepted as a palliative treatment for patients with AGC, and has been confirmed to improve quality of life and prolong survival time. In a number of Asian countries, chemotherapy doublets are frequently used, while in Western countries, triplet regimens are more widely adopted. However, the median survival time, even with contemporary regimens, is typically less than one year (14,26,27). No standard chemotherapy regimen has been established worldwide (28). The V325 trial, a randomized phase III trial, has demonstrated that adding docetaxel to CF significantly improves RR (37 vs. 25%), TTP (5.6 vs. 3.7 months) and OS time (9.2 vs. 8.6 months), but does result in a certain increase in toxicity, including grade 3 or 4 neutropenia (82 vs. 57%), which limits its application in clinical management (14). Further tests have revealed that PF and DF appear similar in efficacy against advanced or recurrent gastric cancer, with different, but acceptable, safety profiles (15,16). PX has begun to emerge in the treatment of AGC, and thymidine phosphorylase is an important enzyme in the progress of capecitabine conversion to 5-FU. In a human colon cancer xenograft model, thymidine phosphorylase was upregulated, and synergy with PX was observed (29). This reveals that the combination of PX may have a coordinated effect. A phase II study reported by Kang *et al* (25) also showed that in 45 AGC Korean patients treated with PX as first-line combination chemotherapy, the tumor RR was 48.9%, the median TTP was 5.6 months and the median OS time was 11.3 months. In addition, grade 3 or 4 adverse events included neutropenia (46.5% of patients), hand-foot syndrome (9.3%), arthralgia (9.3%) and asthenia (4.7%). However, few large-scale randomized trials have been conducted on Chinese patients.

As this study was not a prospective study or large-scale trial, it has evident deficiencies. Due to incomplete medical records and follow-up data, only 36 patients were enrolled, which affected the overall value of the study. However, this retrospective study may also be considered meaningful, as few studies have yet been published concerning PX treatment with a good outcome in AGC patients.

In conclusion, the combination of PX may present as a valuable first-line therapy for advanced or recurrent gastric cancer. We hypothesize that PX may be convenient even in maintenance chemotherapy. Future large-scale studies are urgently required. In addition, for increased survival times and an improved performance status, studies at the molecular biological level, including resistance mechanisms and targeted therapy (for example, antiangiogenic biologicals or trastuzumab in patients positive for HER-2), are likely to be significant issues in the future.

## Figures and Tables

**Table I:** Baseline characteristics of the study population (n=36).

Variables	Value	%
Gender, n		
Male	25	69.4
Female	11	30.6
Age, years		
Median	53.5	
Range	28–75	
Primary site, n		
Esophagogastric junction	6	16.7
Body of stomach	21	58.3
Gastric antrum	2	5.6
Diffuse gastric lesions	7	19.4
Histology, n		
Well-differentiated	0	0.0
Moderately-differentiated	11	30.6
Poorly-differentiated	25	69.4
Surgical history, n		
Yes	8	22.2
No	28	77.8

**Table II:** Cox regression analysis concerning the PFS of AGC patients.

Variables	HR	95% CI	P-value
Gender	0.469	0.186–1.182	0.108
Age	0.842	0.378–1.874	0.674
Primary site	0.786	0.756–4.233	0.226
Surgical history	1.788	0.533–1.161	0.186

PFS, progression-free survival; AGC, advance gastric cancer; HR, hazards ratio; CI, confidence interval.
